# Remyelination therapies are achievable in clinical practice—Commentary

**DOI:** 10.1177/13524585251396267

**Published:** 2025-12-04

**Authors:** Anna Karin Hedström

**Affiliations:** Department of Clinical Neuroscience, Karolinska Institutet, Stockholm, Sweden; Department of Neurology, Karolinska University Hospital, Stockholm, Sweden

In this controversy, Zuroff, Cordano, and Green argue that remyelination therapies are achievable in clinical practice, while Shirani and Stüve emphasize the biological and translational barriers that still impede success. These perspectives reflect the familiar tension between the optimism of biological feasibility and the pragmatism of clinical translation.

The biological rationale for remyelination as a neuroprotective strategy is well supported. In animal models, restored myelin sheaths prevent axonal degeneration and preserve function,^1^ and early human trials show measurable improvements in visual evoked potential (VEP) latency.^2,3^ These findings support the view that the adult human central nervous system retains an intrinsic capacity for repair that can be pharmacologically enhanced.

However, translating this promise into clinical benefit remains challenging. Although phase II trials have demonstrated reduced VEP latency,^2,3^ they have not yet produced meaningful gains in disability or function. Repair capacity appears to decline with age, disease duration, and cumulative axonal loss, and oligodendrocyte precursor cell (OPC) failure may arise from diverse, stage-specific mechanisms. Clinically, a therapy that promotes OPC differentiation in animal models may have limited effect once axonal loss and gliosis predominate. In other words, repair may be biologically feasible, but the therapeutic window is narrower, and the conditions more specific, than initially assumed.

Both contributions agree that the barriers to successful remyelination are as much methodological as biological. Current tools such as VEPs, magnetization transfer imaging, and myelin water fraction are valuable as exploratory or proof-of-mechanism measures, but they are not validated surrogate endpoints for regulatory use.^4^ Without reliable biomarkers, even well-designed studies risk misinterpreting biological effects or underestimating therapeutic potential. Beyond biomarker development, progress will also require more selective enrollment, closer alignment of outcomes with the biology of repair, and more effective drug delivery across the blood-brain barrier.

Remyelination has clinical potential provided that interventions are matched to underlying biology. Experience from Alzheimer’s disease suggests that alignment of patient selection, treatment timing, and outcomes with disease biology is critical for meaningful progress.^5^ Similarly, trials in MS may benefit from stage-specific designs with biomarker enrichment where feasible, by continuing to refine candidate, pathway-specific measures of demyelination and axonal preservation, allowing sufficient treatment duration, and including pathway-specific outcomes alongside clinically relevant measures. Aspects of this approach are already evident in MS studies, although short treatment durations and broad enrollment have often limited the ability to detect repair. Lessons from neurodegenerative trial design may help address these gaps in MS.

So, are remyelination therapies achievable in clinical practice? The biological answer is yes. The practical answer is not yet. The gap between biological feasibility and clinical reality remains. But it is worth recalling that, not long ago, effective disease-modifying therapy also seemed unlikely. While the biological challenge is different, the same commitment that transformed control of acute inflammatory disease activity can help move repair forward.

## References

[1] Irvine KA Blakemore WF. Remyelination protects axons from demyelination-associated axon degeneration. Brain 2008; 131(Pt6): 1464–1477. 18490361 10.1093/brain/awn080

[2] Green AJ Gelfand JM Cree BA , et al. Clemastine fumarate as a remyelinating therapy for multiple sclerosis (ReBUILD): A randomized controlled, double-blind, crossover trial. Lancet 2017; 390: 2481–2489. 29029896 10.1016/S0140-6736(17)32346-2

[3] Brown JWL Cunniffe NG Prados F , et al. Safety and efficacy of bexarotene in patients with relapsing-remitting multiple sclerosis (CCMR One): A randomized, double-blind, placebo-controlled, parallel-group, phase 2a study. Lancet Neurol 2021; 20: 709–720. 34418398 10.1016/S1474-4422(21)00179-4

[4] Mancini M Karakuzu A Cohen-Adad J , et al. An interactive meta-analysis of MRI biomarkers of myelin. Elife 2020; 9: r61523. 10.7554/eLife.61523 PMC7647401 33084576

[5] Pascoal TA Aguzzoli CS Lussier FZ , et al. Insights into the use of biomarkers in clinical trials in Alzheimer’s disease. Ebiomedicine 2024; 108: 105322. 39366844 10.1016/j.ebiom.2024.105322 PMC11663755

